# Sulfated Cellulose Nanofiber Hydrogel with Mucus-Like Activities for Virus Inhibition

**DOI:** 10.1021/acsami.4c17998

**Published:** 2024-11-25

**Authors:** Yanping Long, Mathias Dimde, Julia M. Adler, Ricardo Martin Vidal, Tatyana L. Povolotsky, Philip Nickl, Katharina Achazi, Jakob Trimpert, Benedikt B. Kaufer, Rainer Haag, Chuanxiong Nie

**Affiliations:** † Institute for Chemistry und Biochemistry, Freie Universität Berlin, Takustr. 3, 14195 Berlin, Germany; ‡ Institut für Virologie, Freie Universität Berlin, Robert von Ostertag-Str. 7-13, 14163, Berlin, Germany; § Forschungszentrum für Elektronenmikroskopie und Gerätezentrum BioSupraMol, Freie Universität Berlin, Fabeckstraße 36A, 14195 Berlin, Germany

**Keywords:** mucin-mimetic biopolymeric nanofibers, mucus-like hydrogels, virus binding and inhibition, live-cell imaging, transwell assay

## Abstract

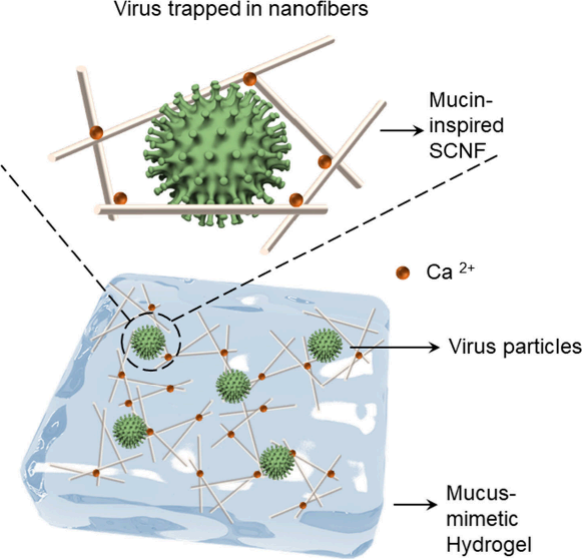

Mucus is the first defense barrier against viruses in the human immune system. Inspired by the mucus structure, we designed a highly sulfated hydrogel to bind viruses and prevent infection of the underlying cells. The hydrogel was formed by gelation of sulfated cellulose nanofiber (SCNF) with Ca^2+^. SCNF exhibited a mucin-like nanofiber structure with high numbers of sulfated groups. Based on the electrostatic interactions with a virus, SCNF could efficiently inhibit herpes simplex virus-1 (HSV-1) infection with a half-maximal inhibitory concentration (IC_50_) of 0.43 μg/mL, which is comparable to that of heparin (IC_50_ = 0.30 μg/mL). Benefiting from the multiporous structure and sulfate groups, the Ca^2+^-SCNF hydrogel could efficiently trap HSV-1 and inhibit the virus from attacking the underlying cells in a transwell model. Furthermore, SCNF also inhibited SARS-CoV-2 infection in a similar experimental setting. By integrating the advantages of high and broad-spectrum virus inhibitory activity as well as low toxicity, it is believed that the Ca^2+^-SCNF hydrogel can promote the development of highly biocompatible and efficient antiviral material with “binding and inhibition” capability and other diverse strategies.

## 1 Introduction

Viruses are ongoing challenges to public health, and according to the World Health Organization, virus infections are among the leading causes of mortality worldwide.^1^ For instance, the recent outbreak of coronavirus disease 2019 (COVID-19) caused by the severe acute respiratory syndrome coronavirus 2 (SARS-CoV-2) has caused more than 6 million deaths and had a massive impact on the global economy and employment situation.^2,3^ At the same time, other viruses, such as herpes simplex virus-1 (HSV-1), are ubiquitous in the human populations and continue to pose a substantial health burden.^4^ Worse still, the virus is still undergoing genetic mutations, leading to an unpredictable evolution of surface proteins or antigens and, consequently, the development of resistance to therapeutics and vaccines.^5^ Innovative strategies to inhibit virus infection with broad-spectrum activity are urgently needed in biomedical science.^6−9^

**Scheme 1:**
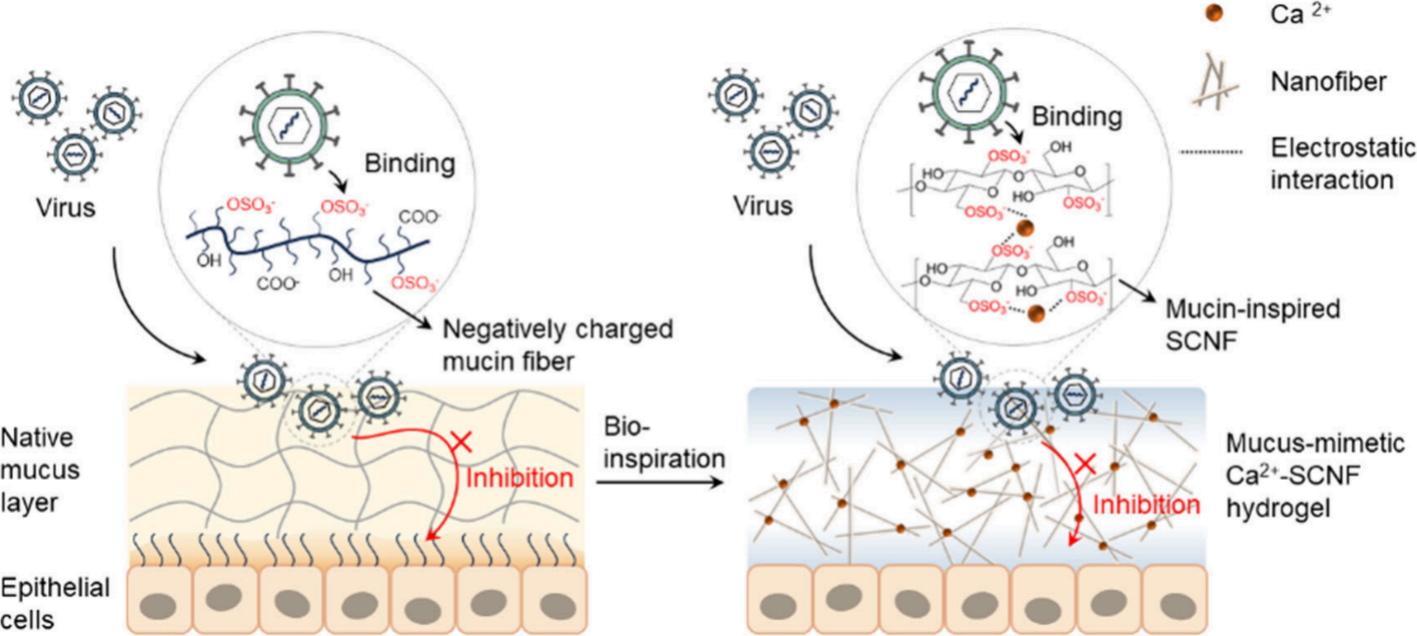
Schematic Illustration of Proposed Antiviral Mechanism of Mucin-Inspired Sulfated Cellulose Nanofibers (SCNF) by Electrostatic Interaction between Virus Glycoproteins and Sulfate Groups in SCNF The addition of Ca^2+^ facilitates the gelation by increasing intramolecular/intermolecular interactions between SCNF chains and forms hydrogels. The synthesized Ca^2+^-SCNF hydrogel could mimic a native mucus layer to trap a virus and prevent the virus from penetrating the gel so that the virus cannot infect the underlying epithelial cells.

In the human immune system, mucus plays an important role in preventing cellular contact with viruses and other pathogens. It is a highly hydrated polymer network consisting of ultrahigh molecular weight glycoproteins named mucins.^10,11^ Located on N-acetylglucosamine, galactose, mannose, sialic acid sugars, and sulfated glycans on mucins are key contributors to interacting with viruses. The sulfated glycans, including chondroitin sulfate, heparan sulfate, and keratan sulfate are important glycosaminoglycans and are of interest for virus interaction.^12^ Besides, the thiol groups in mucin can allow the formation of a dynamic gelatinous structure lying above the epithelium to create a biological interface from the environment (left in Scheme 1).^13,14^ Therefore, when viruses intrude the human body, they are commonly trapped in the mucus layer by electrostatic interactions; their entries are inhibited and then cleared by cilia beating before starting the infection cycle in the host.^15^

Inspired by the importance of mucin in defending virus infection, efforts have been devoted to develop mucin-mimetic nanostructures as binding decoys to interfere with the viral entry process, for example, linear/dendritic polymers,^16^ phage capsid,^17^ protein nanofibrils,^18^ wormlike dendronized polysulfates,^19^ and brush polymer with sialyl oligosaccharide.^20^ This is because viral entry into host cells is considered to be the first and one of the most essential steps for virus infection, which are mostly mediated by the multivalent interactions between viral spike proteins and the receptors on host cells.^21^ To take advantage of multivalent interaction and the beneficial characteristic of a mucus entrapping virus, hydrogels have been put forward with advantages of easy administration, minimal influence on body health, and intimate contact between antiviral agents and the virus surface.^22^ For example, glycosylated hydrogels with mesh could trap norovirus through a “caged polyvalent effect”,^23^ and sulfated dendritic polyglycerol hydrogels were able to bind and trap HSV-1.^24^

Therefore, in this study, we combined the morphological and structural characteristics of the mucus hydrogel. In the beginning, a mucin-like sulfated cellulose nanofiber (SCNF) was designed via the direct sulfation of cellulose in a deep eutectic solvent, and its interaction with virus particles was investigated in vitro (Scheme 1). The sulfated nanofiber could bind to HSV-1 and SARS-CoV-2 virus particles and prevent their infection as demonstrated by cellular infection assays. The SCNF exhibits mucin-like properties, including fibrous nanostructure and functional sulfate groups to defend against pathogens (HSV-1 and SARS-CoV-2) and could work as an important component of the hydrogel. By adding Ca^2+^ into SCNF solution, formation of mucus-mimetic hydrogels was achieved as shown by rheology studies. The synthesized 0.01 M Ca^2+^-SCNF hydrogel showed a *G*′ value of ∼2.17 Pa at 1 Hz and presents viscoelastic shear-thinning materials, which is at the same level of mucus (*G*′ value of 1–10 Pa in concentration dependent manner) showing a viscoelastic property comparable to the biological systems.^25,26^ Furthermore, the ability of the Ca^2+^-SCNF hydrogel to bind and trap viruses was verified via a transwell infection model.

## 2 Experimental Section

### 2.1 Materials

Sulfamic acid, urea, carboxymethyl cellulose (CMC), sodium bromide (NaBr), fluorescein thiocyanate (FITC), and heparin sodium salt were purchased from Sigma-Aldrich. Microcrystalline cellulose (MCC) was purchased from Macherey-Nagel GmbH & Co. KG. 2,2,6,6-Tetramethylpiperidine-1-oxyl (TEMPO) and sodium hypochlorite (NaClO) were purchased from Carl Roth GmbH + Co. KG. The deionized water used is purified using a Millipore water purification system with minimum resistivity of 18.0 MΩ cm.

### 2.2 Preparation of Sulfated Cellulose Nanofibers (SCNF) Derived from Microcrystalline Cellulose (MCC)

The sulfamic acid and urea were mixed together using a magnetic stirrer at 90 °C until a clear solution was obtained. Then, cellulose was added and was uniformly immersed in the above-mentioned solution. Then, the temperature of the system was increased to 160 or 120 °C and proceeded for 2 h. The reaction mixture was then removed from the oil bath and cooled to room temperature. The reaction was finally terminated by the addition of excess water, followed by centrifugation and washing with water. Then, saturated NaOH solution was added to neutralize the sulfated cellulose nanofibers, followed by centrifugation, dialysis, and lyophilization.

### 2.3 Labeling of SCNF-2 and Cellular Uptake of Ca^2+^-SCNF-FITC Hydrogel

The SCNF-2 was labeled with FITC for fluorescent microscope observation. For the labeling, FITC was dissolved in DMF to 2.5 mg/mL; then, SCNF-2 and triethylamine were added and stirred in the dark for 24 h in 80 °C. Then, the SCNF-FITC was separated, followed by centrifugation, dialysis, and freeze-drying. Finally, 100 μL of CaCl_2_ solution dissolved in deionized water (0.1 M) was added to 900 μL of SCNF-FITC solution (10 mg/mL) using pipettes and mixed via a vortex mixer to form hydrogel with final Ca^2+^ concentration of 0.01M.

For the cellular uptake experiment, the Ca^2+^-SCNF-FITC hydrogel was added onto Vero E6 cells (ATCC CRL-1586) and incubated at 37 °C for 2 h. Afterward, Vero E6 cells were stained with Hoechst 33342. Then, formaldehyde was added to fix the cells, and the fluorescent images for cellular uptake were acquired on Leica SP8 confocal laser scanning microscopy (CLSM).

### 2.4 Characterization of SCNF

Elemental composition determination was performed on a Vario EL CHNS element analyzer by Elementar Analysensysteme GmbH (Langselbold, Germany). SEM testing was carried out using a Hitachi SU8030. Transmission electron microscopy (TEM) measurements were carried out using a TALOS L120C electron microscope (Thermo Fisher Scientific Inc., Waltham, Massachusetts, USA). For this measurement, droplets of the sample solution (5 μL) were applied on hydrophilized Formvar-supported carbon-coated copper grids (400 mesh) for 60 s and stained with uranyl acetate solution for 45 s (negative stain). Hydrophilization was achieved beforehand by a 60 s glow discharging in an Emscope SC 500 device at 20 mA. The supernatant fluid was removed by blotting with a filter paper. XPS experiments were performed with an EnviroESCA spectrometer (SPECS Surface Nano Analysis GmbH, Berlin, Germany), equipped with a monochromatic Al Kα X-ray source (excitation energy = 1486.71 eV) and a PHOIBOS 150 electron energy analyzer. SEM images were obtained by using a SU8030 scanning electron microscope (Hitachi).

### 2.5 HSV-1 Inhibition Performance of Heparin and SCNFs

The HSV-1 inhibition performances of heparin and SCNFs were investigated via a preinfection inhibition assay and plaque reduction assay. The GFP expressing HSV-1 used in this study was generated by the Institute of Virology at the Free University of Berlin, using a reverse genetics system. To this end, the HSV-1 F-strain bacterial artificial chromosome (pYEbac102 kindly provided by Y. Kawaguchi, University of Tokyo, Japan) was modified by inserting GFP into the BAC cassette under the control of the immediately early cytomegalovirus (CMV) promoter.^27^ GFP expression was confirmed by fluorescent microscopy with green fluorescence colocalized with cytopathic effects characteristic of HSV-1 replication. Virus stocks were subsequently produced and titrated on the Vero cells.

For the preinfection inhibition assay, Vero E6 cells were seeded into 96-well plates; after being washed with phosphate-buffered saline (PBS), the cells were incubated with SCNFs of different concentrations for 45 min, followed by adding 10 μL of 10^6^ PFU/mL HSV-1. Then, after 24 h incubation and fixation, Hoechst 33342 was used to stain cell nuclei, and fluorescent images were acquired on a Zeiss Axio Observer Z1 microscope (ZEISS, Germany). The total cells and infected cells were counted via ImageJ, and the infection was then estimated by the ratio of infected cells in total cells.

The half maximal inhibitory concentration (IC_50_) was determined by plotting the infection ratio of Vero E6 cells to the compound concentration. Curve fitting was done using Graphpad Prism 9 with [Agonist] vs normalized response [Y = 100X/(EC50+X)].

For the plaque reduction assay, HSV-1-GFP (10^3^ PFU/mL) was pretreated with SCNFs of different concentrations and heparin, and then, the inoculum was incubated with Vero E6 cells to assess virus binding. Afterward, the cells were washed with PBS to remove unbound virions. Then, the cells were cultured for 72 h with overlay medium for plaque formation. Finally, the plaque reduction ratios were calculated by comparing the PFU of the sample-treated virus solution and the nontreated virus solution as follows:
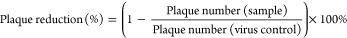


### 2.6 Formation and Characterization of Hydrogel

For the formation of Ca^2+^-formed hydrogel, 100 μL CaCl_2_ dissolved in deionized water (0.1 M, 0.5 M, 1 M) was added to 900 μL SCNF-2 aqueous solution (10 mg/mL) using pipettes and mixed via vortex mixer, so the Ca^2+^-SCNF hydrogel was obtained with Ca^2+^ concentration being 0.01 M, 0.05 M, and 0.1 M.

For the characterization of the Ca^2+^-SCNF hydrogel, oscillatory rheology experiments were taken to indicate the mechanical properties of the hydrogel. All the rheology data of hydrogel samples were characterized by Malvern Instruments Kinexus, and the average normal force of estimate was 0.1 N at 25 °C. The data were analyzed by an oscillatory frequency sweep strain-controlled test with 1% strain, and the reported storage modulus (*G*′) and loss modulus (*G*″) were picked at 1 Hz. The viscoelastic behavior was also analyzed by recording the response of viscosity to the shear rate, which was examined in continuous flow experiments with a linearly ramped shear rate from 0.01 to 100 s^–1^.

The microstructures of the hydrogels were investigated with SEM. The fabricated hydrogel was frozen by liquid nitrogen, and then, the freeze-dryer was used to remove the water inside. Then, the gold coating was performed, followed by SEM high-resolution imaging, enabling the detailed visualization of the hydrogel surface at the nanometer scale.

### 2.7 HSV-1-GFP Binding and Interaction of Ca^2+^-SCNF Hydrogel

To test the HSV-1 inhibition of the hydrogel, a plaque assay was first used. Here, 135 μL of cell culture medium was added to 96-well plates. Hydrogels were incubated with HSV-1 at still conditions for 30 min. Then, 15 μL of hydrogel-incubated HSV-1-GFP supernatant solution was added, and 10-fold dilution was performed. Meanwhile, 15 μL 0.01 M Ca^2+^-SCNF hydrogel precipitate was taken, and 10-fold dilution was performed. Then, these dilutions were transferred to the 24-well plates with Vero E6 cells at ∼100% confluency and were shaken slightly for 45 min. Finally, the inoculum was removed, and an overlay medium was added for plaque formation after being washed. Then, the number of plaques were counted, and the PFU of a hydrogel-treated virus supernatant and PBS-treated virus solution were calculated and compared.

To further verify that the hydrogel could efficiently prevent the virus from reaching the cells beneath, the HSV-1-GFP infection with the hydrogel in a transwell model was used. At first, Vero E6 cells were seeded in 24 well plates. Then, 3 μm-transwells were placed onto the cells, followed by adding hydrogel into the transwell inserts with homogeneous distribution and complete coverage. In the meantime, the same volume of PBS was added as a comparison sample. Then, HSV-1-GFP was added into the transwell and kept for 3 days. Finally, the cells were washed with PBS and fixed by 4% formaldehyde. Then, the cell nuclei were stained with Hoechst 33342, and fluorescent images were acquired on a Zeiss Axio Observer Z1 microscope (ZEISS, Germany).

## 3 Results and Discussion

### 3.1 Synthesis of Mucin-Like Sulfated Cellulose Nanofibers (SCNF) Derived from Microcrystalline Cellulose (MCC)

**Figure 1:**
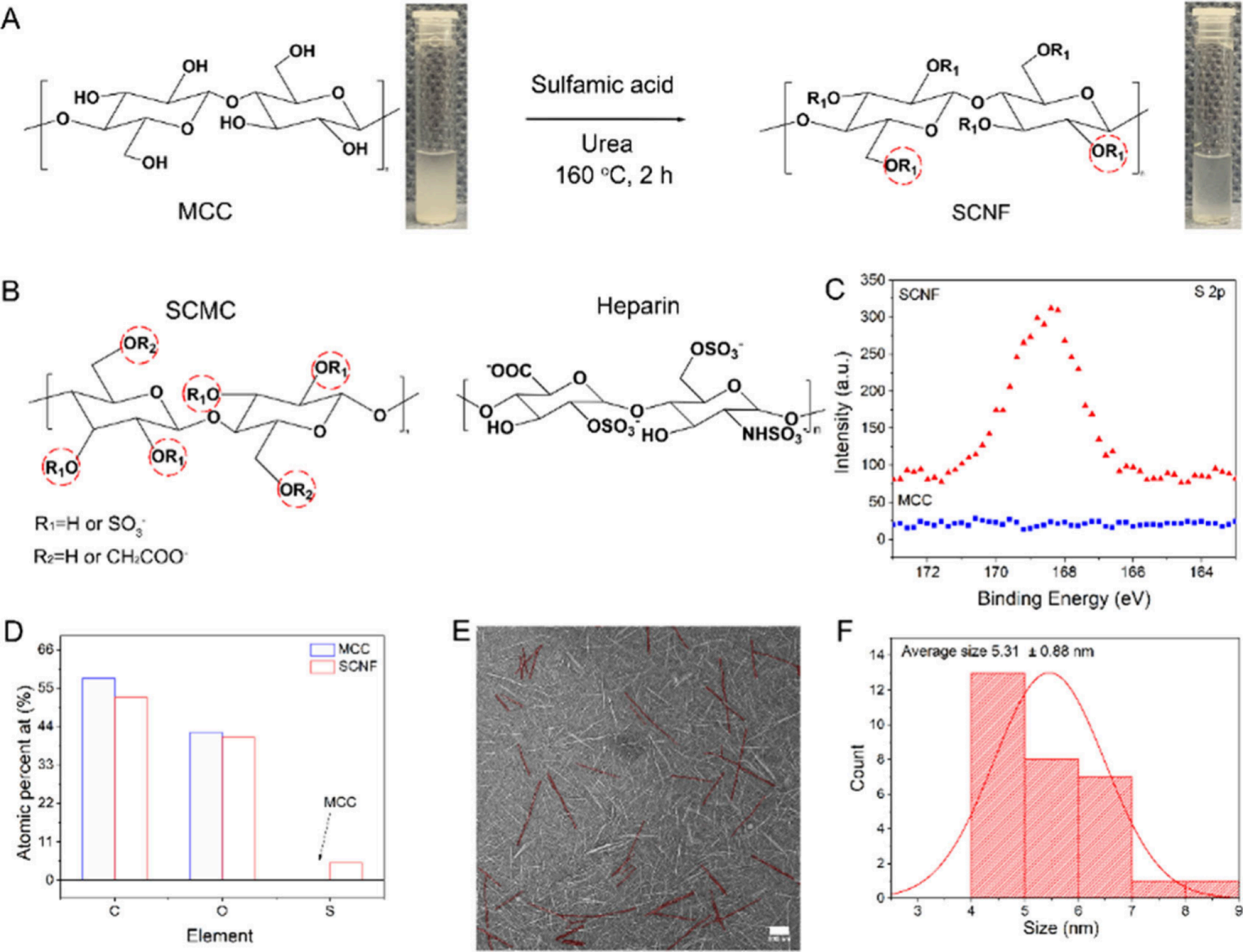
(A) Preparation process of sulfated cellulose nanofibers (SCNF) derived from microcrystalline cellulose (MCC) sulfation. The insets are digital photographs of the MCC and SCNF. (B) Chemical structure of sulfated carboxymethyl cellulose (SCMC) and heparin. (C) Highly resolved S 2p XPS spectrum for MCC and SCNF-2. (D) Elemental content of C, O, and S for MCC and SCNF-2 derived from quantified XPS survey. (E) TEM and (F) corresponding average width of SCNF-2. For a better view, the brightness of the original image was increased. Scale bar = 100 nm.

SCNF was synthesized according to a report by Sirviö et al., as shown in Figure 1A.^28^ Briefly, a deep eutectic solvent composed of sulfamic acid and urea was prepared by heating at 90 °C, among which urea helped to liquidize sulfamic acid for the sulfation reaction with cellulose. Then, the MCC was added to the urea-sulfamic acid liquid mixture for 2 h at 160 or 120 °C. The reaction was ceased by cooling to room temperature, and the product was washed with water and pelleted by centrifugation. The counterion of sulfate groups was exchanged to sodium by adding NaOH to the SCNF solution to a final pH of 8.0. Finally, the SCNF was purified by dialysis against distilled water, which yielded a highly water-soluble polymeric nanostructure as shown in Figure 1A. Meanwhile, as a highly sulfated derivative of heparan sulfate, heparin has been widely used as an antiviral, and its chemical structure is shown in Figure 1B, presenting a similar structure as SCNF and being used for comparison in later studies.

Moreover, SCNFs with different degrees of sulfation (DS) were obtained by changing the molar ratio of sulfamic acid/MCC and reaction temperature, as shown in Table 1. Specifically speaking, when reacted with 6 equiv of sulfamic acid at 120 °C, the obtained SCNF-4 has the DS of only 6%. In order to optimize the SCNF reaction, we increased the reaction temperature to 160 °C to obtain SCNF-3 (DS = 13%). Besides, the sulfamic acid/cellulose ratio also plays an important role in sulfation efficiency; compared to DS of SCNF-3, when reacted with 10 equiv of sulfamic acid at 160 °C, the produced SCNF-2 had the highest DS (31%) among all samples. In conclusion, the high sulfamic acid amount and high reaction temperature are necessary to achieve high DS.

**Table 1:** Reaction Condition and Characterization of SCNFs, SCMC, OCNF, Heparin, and Original Celluloses

Sample	Sulfamic acid (eq. to cellulose)	Urea (eq. to cellulose)	Time (h)	Temperature (°C)	Degree of sulfation^a^	Zeta potential (mV)	IC_50_ (HSV-1) (μg/mL)^b^
SCNF-1	4	16	2	160	0.09 ± 0.01	–35.74 ± 1.44	27.37
SCNF-2	10	20	2	160	0.31 ± 0.10	–38.59 ± 2.61	0.43
SCNF-3	6	12	2	160	0.13 ± 0.05	–35.28 ± 4.05	15.25
SCNF-4	6	12	2	120	0.06 ± 0.01	–31.96 ± 1.22	391.80
SCMC	6	12	2	160	0.17 ± 0.04	–64.34 ± 2.85	2.58
OCNF	–	–	–	–	0	–34.86 ± 3.02	>1000
Heparin	–	–	–	–	0.98 ± 0.08	–19.45 ± 2.27	0.30
MCC	–	–	–	–	0	–8.74 ± 1.09	>1000
CMC	–	–	–	–	0	–24.45 ± 0.95	>1000

a Degree of sulfation (DS) determined by elementary analysis.

b IC50 determined by preinfection inhibition assay.

The sulfation of MCC was compared to commercially available counterparts, carboxymethyl cellulose (CMC), for which the hydroxyl groups have been partially substituted with carboxymethyl groups (degree of substitution 1.2), as shown in Figure 1B. Compared with MCC, the sulfation of CMC was more challenging, proved by the fact that the sulfated CMC showed only a degree of sulfation of 0.17 at the same reaction condition. Since the hydroxyl groups have also been partially substituted by carboxymethyl groups, it is reasonable that the degree of sulfation is lower for CMC than that of MCC under the same reaction condition. However, sulfated CMC exhibited more negative charges than SCNF-2, due to the existence of carboxyl groups. It is also noticed that the sulfated CMC has a lower viscosity than SCNF at the same concentration in Figure S1, which is correlated with carboxymethyl side groups partially preventing hydrogen bonds between the cellulose molecular chains.

X-ray photoelectron spectroscopy (XPS) and Fourier-transform infrared spectroscopy (FTIR) were applied to characterize the structures of SCNFs. As seen from XPS analysis results, shown in Figure 1C and D, the S 2p signal was observed for SCNF, while no S 2p signal for pure MCC could be detected. The elemental content of sulfur in SCNF was approximately 5 at. %. In FTIR, the asymmetric S=O and symmetric vibrations of sulfate group were noticed at 1228 and 807 cm^–1^, respectively, proving the successful sulfation of MCC.^29^ The carbonyl vibration at 1641 cm^–1^ is worth mentioning, which may originate from a carbamate group caused by a side reaction between sulfamic acid and urea as shown in Figure S2.^30^

Then, the nanostructure of SCNF-2 was investigated by transmission electron microscopy (TEM) and scanning electron microscopy (SEM). In Figure 1E and F, SCNF-2 exhibits characteristic rod-like morphology and shows a uniform distribution with an average width of 5.3 nm and with length of 100–200 nm. The formation of uniformly sized nanofibers is likely attributed to the high charge density. This high charge density induces electrostatic repulsion between the nanofibrils and improves water penetration into the fibers.^31^ We also compared the morphology of SCNF-2 with that of an oxidized cellulose nanofiber (OCNF) prepared via the 2,2,6,6-tetramethylpiperidinyloxyl (TEMPO)-mediated method as shown in Figure S3. It was noticed that SCNF-2 had a similar morphology and a similar appearance in aqueous solution to OCNF. However, comparing the synthesis, sulfation is much easier to achieve as it spares the efforts of controlling the reaction pH, and the reaction was more straightforward than TEMPO oxidation. It is therefore believed that SCNF can be a substitute to the OCNF for other applications; however, its performance in different scenarios should be further investigated.

### 3.2 HSV-1 Inhibition Performance of Heparin, OCNF, SCNFs, and SCMC

HSV-1 is reported to strongly interact with mucus,^32^ and HSV-1 infection toward host cells can be prevented by heparan sulfate, a natural polysaccharide as an essential component in mucus. Known as the highly sulfated derivative of heparan sulfate, heparin is widely used in biomedical research and, therefore, was utilized as the control group to compare the antiviral effect. In this study, we used genetically green fluorescent protein (GFP) modified HSV-1^27,33^ as a model to study inhibitory activities of SCNFs as infected cells would express GFP and subsequently could be detected. Prior to assessing the virus inhibitory effect, a cell viability assay with cell counting kit-8 (CCK8) was performed to study a possible cytotoxic effect of SCNFs on Vero E6 cells. In Figure S4, SCNFs and heparin show no effect on the viability of Vero E6 cells up to a concentration of 1 mg/mL.

**Figure 2:**
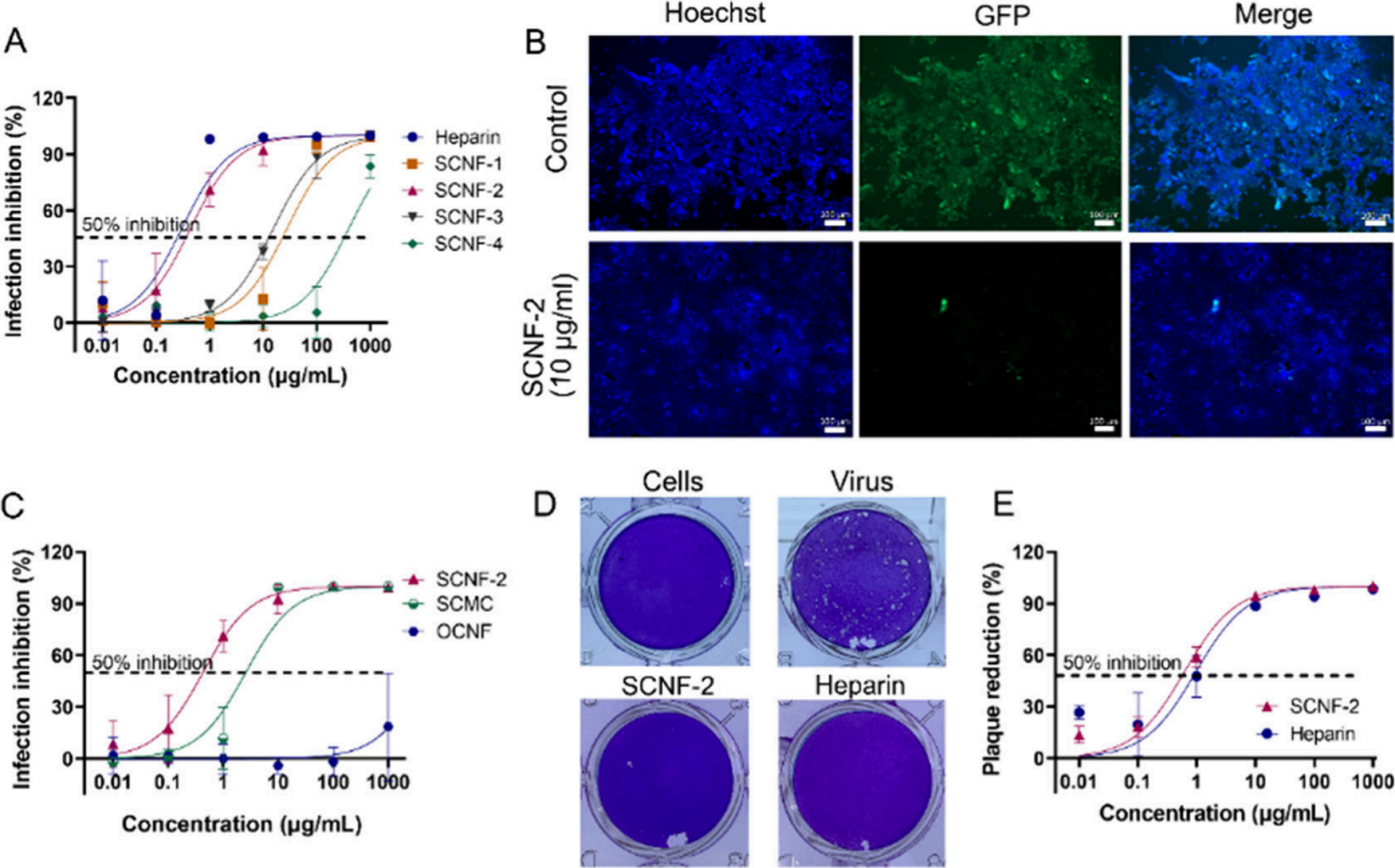
(A) Preinfection inhibition ratio for the SCNF samples and heparin at different dose. Values are presented as mean ± SD, *n* = 3. (B) Microscope images showing the HSV-1-GFP-infected cells incubated with 10 μg mL^–1^ SCNF-2. Scale bar = 100 μm. (C) Infection inhibition of HSV-1-GFP by SCNF-2, SCMC, and OCNF at different concentrations (MOI 0.1). Values are presented as mean ± SD, *n* = 3. (D) Representative images for the reduction of plaque formation of HSV-1-GFP for inhibitors at a concentration of 10 μg mL^–1^. (E) Plaque-reduction ratios for the inhibitors at different concentrations. Values are presented as mean ± SD, *n* = 3.

We first evaluated the virus inhibitory activity in a preinfection inhibition setting. Herein, we preincubated the cells with the samples for 45 min and then infected the cells with HSV-1-GFP at a multiplicity of infection (MOI) of 0.1 in the presence or absence of the samples. The infection was then analyzed after 24 h by fluorescent microscopy, as shown in Figure 2A and B, and all cells expressing GFP were regarded as infected and being detected. Sulfation degree-dependent antiviral activity was observed; SCNF-2 with the highest sulfation degree and maximal negative charges showed the best inhibitory effect with a half-maximal inhibitory concentration (IC_50_) of 0.43 μg/mL, which was comparable to IC_50_ value of heparin (IC_50_ = 0.3 μg/mL). As shown in Figure 2B and Figure S5, the negligible HSV-1-GFP signal in SCNF-2-treated cells and heparin-treated cells indicates that SCNF-2 and heparin could effectively bind and prevent virus infection. MCC itself did not show any inhibition of HSV-1-GFP; neither did CMC, carrying negatively charged carboxyl groups, as shown in Figure S6.

In Figure 2C, the OCNF (with carboxylic acid groups) does not inhibit HSV-1 infection effectively, proving that the negatively charged sulfate groups are an essential part in electrostatically interacting with HSV-1. Then, we also investigated the HSV-1 inhibitory activities of sulfated cellulose derivatives (SCNF-2, SCMC). SCNF-2 (DS = 0.31) and SCMC (DS = 0.17) both showed inhibition performance against HSV-1-GFP. SCNF-2 (with sulfate groups) outperformed SCMC (with carboxylic acid groups) for virus inhibition, although SCMC carried more negative charges because of carboxylic acid groups. In summary, although SCMC and OCNF carried more negative charges mainly due to the abundant carboxylic acid groups than SCNF, they still showed limited HSV-1 inhibition capability, meaning the sulfate group plays an irreplaceable role in contributing to binding HSV-1 in the cellulose system.

Furthermore, the virus inhibitory activity was also confirmed by the plaque reduction assay (PRA). Herein, HSV-1-GFP was first incubated with the samples and then inoculated onto cells for the infection. Afterward, the cells were washed and overlaid with an Avicel overlay medium to assess the development of plaques. By comparing the number of plaques with the control, dose-dependent inhibition curves were obtained as shown in Figure 2D and E. Herein, the potent virus inhibitory activity of SCNF-2 was again proven with an IC_50_ (PRA) of 0.61 μg/mL, which was at the same level as heparin (IC_50_ (PRA) 0.96 μg/mL).

For further studies, SCNF-2 was selected over heparin after several considerations and is a promising alternative. As for biocompatibility, SCNF biopolymers being made from cellulose are generally nontoxic, which is comparable to naturally occurring heparin. Nevertheless, SCNFs are derived from abundant and renewable cellulose, making them more cost-effective and easier to produce in large quantities with costs of less than 1 euro per gram. Additionally, heparin itself lacks hydrogel-formation capability, and complicated chemical modification is required for gelation.

### 3.3 Fabrication and Characterization of Mucus-Mimetic Ca^2+^-SCNF Hydrogel

**Figure 3:**
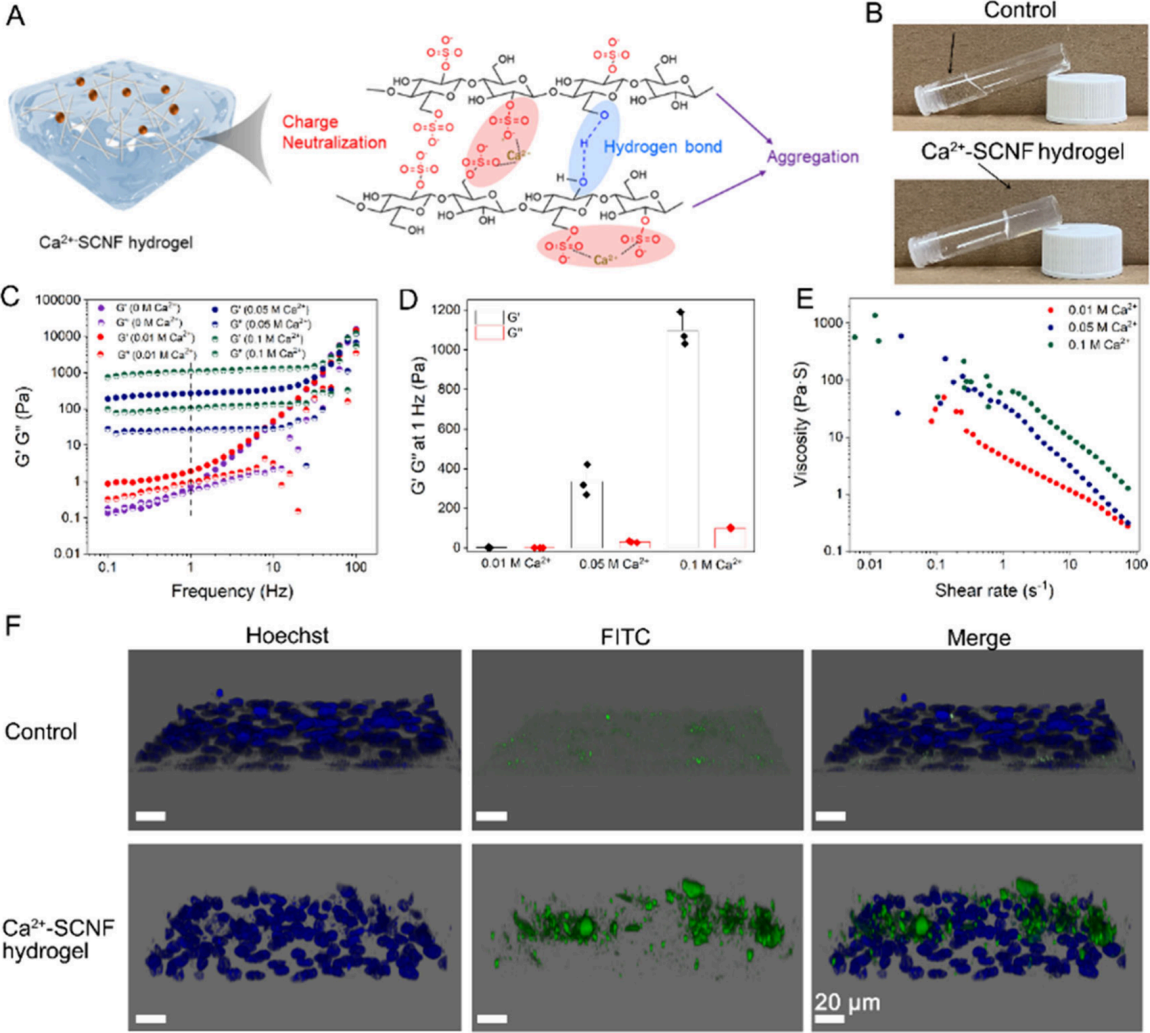
(A) Schematic illustration of the Ca^2+^-SCNF gel formation by Ca^2+^ mediated nanofibers aggregation via neutralizing sulfate groups’ negative charge. (B) Digital photographs of SCNF solution and Ca^2+^-SCNF hydrogel. (C) Frequency sweep ramp performances of the SCNF solution and Ca^2+^-SCNF hydrogel. (D) The *G*′ and *G*′’ values of Ca^2+^-SCNF hydrogels at 1 Hz. Values are presented as mean ± SD, *n* = 3. (E) Shear-rate-dependent viscosity moduli of the Ca^2+^-SCNF hydrogels. (F) Images of Vero E6 cells after 2 h incubation without and with Ca^2+^-SCNF hydrogel, in which FITC signal (in green) stands for hydrogel and Hoechst signal (in blue) stands for cell nuclei. Scale bar: 20 μm.

As SCNF-2 exhibited the best antiviral ability among SCNFs, we investigated further its ability to make a mucus-mimicking hydrogel. By mixing Ca^2+^ with SCNF-2 solutions via a vortex mixer, homogeneous and transparent Ca^2+^-SCNF hydrogel was obtained as shown in Figure 3A and B. It is hypothesized that additional metal ions affected ionization of sulfate groups in aqueous solutions, forming sulfate-Ca^2+^ conjugates that are poorly soluble in water. Moreover, the neutralization of negative charges by Ca^2+^ can alleviate the intermolecular repulsive interactions between SCNF chains to form the hydrogels, which was supported by the observation that adding H^+^, K^+^ or even Na^+^ also triggered the formation of SCNF hydrogels, as shown in Figure S7; Ca^2+^, as a divalent metal ion, may bridge more than one sulfate group to induce charge neutralization and hydrogel formation.

Next, we studied rheological properties of hydrogels with the same content of SCNF (1 wt %), but different Ca^2+^ concentrations (0 0.01, 0.05, and 0.1 M, respectively). The oscillatory shear on frequency sweep strain test at a constant strain was performed over the frequency range from 0.1 to 100 Hz as shown in Figure 3C. For these Ca^2+^-SCNF hydrogels, the elastic storage modulus (*G*′) was evidently predominant over viscous loss modulus (*G*′’), indicating that these gels had higher elastic energy storage than energy dissipation, being indicative of gelation. To simplify the shear modulus graph, the *G*′ value at 1 Hz is shown in Figure 3D, which was directly related to the hydrogel stiffness. We also compared the *G*′ value of pure SCNF (∼0.62 Pa) with that of Ca^2+^-SCNF hydrogels (∼2.17–335.37, and ∼1096 Pa for 0.01 0.05, and 0.1 M, respectively). The increased *G*′ value suggested the successful gelation after adding Ca^2+^; meanwhile, along with increase of Ca^2+^ concentration, the *G*′ value increase representing higher hydrogel stiffness.

The hydrogel stiffness could influence biocompatibility, in vivo performance, stability, and flexibility from several perspectives below. The 0.01 M Ca^2+^-SCNF hydrogel with *G*′ of 2.17 Pa maybe perform better in terms of adhesion to mucosal surfaces and flexibility as well as conforming to irregular surfaces, but might lack mechanical stability in dynamic environments. On the contrary, the hydrogel with *G*′ of 1096 Pa might possess high stability but lower flexibility, which might limit application to areas requiring more conformability. In conclusion, the hydrogel with *G*′ of 2.17 Pa is soft and closely mimics the natural mucus layer (*G*′ value of 1–10 Pa in concentration dependent manner); therefore, it is preferentially used in following virus-related tests and was proven to be stable over 7 days in Figure S8.

Scanning electron microscopy (SEM) was used to visualize the microstructure of the Ca^2+^-SCNF hydrogel. In Figure S9, both the Ca^2+^-SCNF (0.01 M) hydrogel and Ca^2+^-SCNF (0.1 M) hydrogel showed three-dimensional network structures with numbers of pores. It was noticed that the compactness enhanced along with the increase of Ca^2+^ concentration; this was caused by the increased nanofiber chain aggregation by Ca^2+^, which was in line with rheology results.^34^ Furthermore, the viscosity variations of the hydrogels at increased shear rate were investigated by a shear rate ramp test. As shown in Figure 3E, we noticed a negative correlation between viscosities and shear rate for these hydrogels. The hydrogels showed elastic behaviors under low shear strain, whereas they possessed viscous liquid naturez under high shear strain, being indicative of the viscoelastic shear-thinning materials like dynamic mucus hydrogel.^35^

We also attempted to mimic biological conditions in which mucus forms a stable barrier above epithelial cells and better achieve virus “binding and prevention” function. Fluorescein isothiocyanate (FITC) was utilized to label the Ca^2+^-SCNF hydrogel, followed by incubation with Vero E6 cells for 2 h. In Figure 3F, the hydrogel represented by a green FITC signal is located above the cells, and no cellular uptake was noticed, similar to mucus functioning on the top layer of the mucosal barrier.

### 3.4 HSV-1 Binding and Trapping of Ca^2+^-SCNF Hydrogel

**Figure 4:**
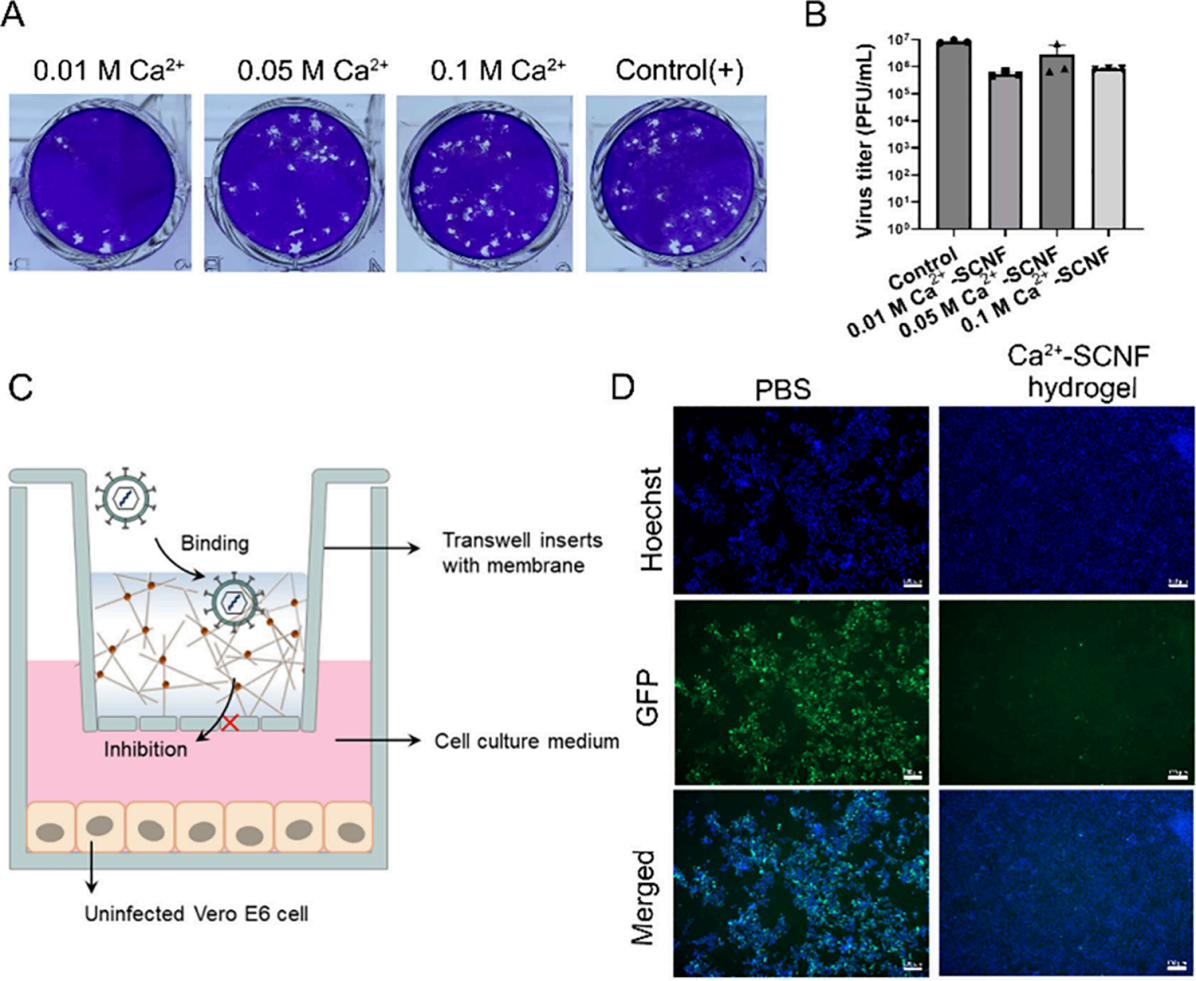
(A) Representative images for plaque formation of HSV-1 when incubated with Ca^2+^-SCNF hydrogel inhibitor. (B) Titer of Ca^2+^-SCNF hydrogel-treated HSV-1 supernatant. Values are presented as mean ± SD, *n* = 3. (C) Schematic illustration of the transwell insert setup during the HSV-1 infection experiment. The hydrogel was added and kept above the transwell membrane, and the Vero E6 cell was attached on the plates under 0.3 μm membrane. (D) Immunofluorescent images for the HSV-1-infected cells with and without 0.01 M Ca^2+^-SCNF hydrogel. Scale bar = 100 μm.

The HSV-1 binding of the Ca^2+^-SCNF hydrogel was studied by a virus adsorption assay. Here, the hydrogels were incubated with HSV-1-GFP for 30 min; then, the virus in the supernatant was titrated. The virus binding capability was evaluated by comparing the virus titers of hydrogel-treated and PBS-treated virus solutions as shown in Figure 4A and B and Figure S10. Compared to the PBS-treated group, a lower virus titer in the hydrogel-treated group and detected virus from hydrogel precipitate indicate that the virus was absorbed by the Ca^2+^-SCNF hydrogel. We also noticed that generally the virus binding capability of hydrogels decreased along with the Ca^2+^ concentration increase, likely a result of differences in the virus diffusion behavior in hydrogels with different stiffnesses. The rheological and SEM investigations demonstrate that the hydrogels became more compact and stiffer with increasing Ca^2+^ levels, which made the penetration and diffusion of virus particles more difficult.

To further demonstrate that the Ca^2+^-SCNF hydrogel can be applied to bind and prevent the virus from infecting cells underneath, we utilized a transwell model to study HSV-1-GFP penetration in the Ca^2+^-SCNF hydrogel (Figure 4C). Herein, the hydrogels were applied in a 3 μm membrane above the cells, whereas the virus was applied on the gel, so only free virus can pass through membrane and infect cells. In Figure 4D, nearly all the cells were infected in the control group after 48 h of incubation, and the cytopathic effect caused by HSV-1 infection led to a dissociation of the cell monolayer. In contrast, only very few infected cells were detected for the Ca^2+^-SCNF hydrogel-treated HSV-1 group, suggesting virus particles were trapped in the mucus-mimetic hydrogel. Moreover, the Ca^2+^-OCNF hydrogel with a carboxylic acid group barely restrained virus infection as shown in Figure S11, which eliminates the influence of hydrogel networks on antiviral activity, being consistent with the conclusion of strong binding between a sulfate-like structure and HSV-1 surface glycoprotein.

Meanwhile, Video S1, Video S2, and Video S3 show the appearance of the first infected cells at 6 h of infection for the control. However, for the Ca^2+^-SCNF hydrogel, the first infected cells were noticed after only 20 h, indicating an impaired diffusion of virus particles through the gel.

### 3.5 Inhibitory Activity of against SARS-CoV-2

**Figure 5:**
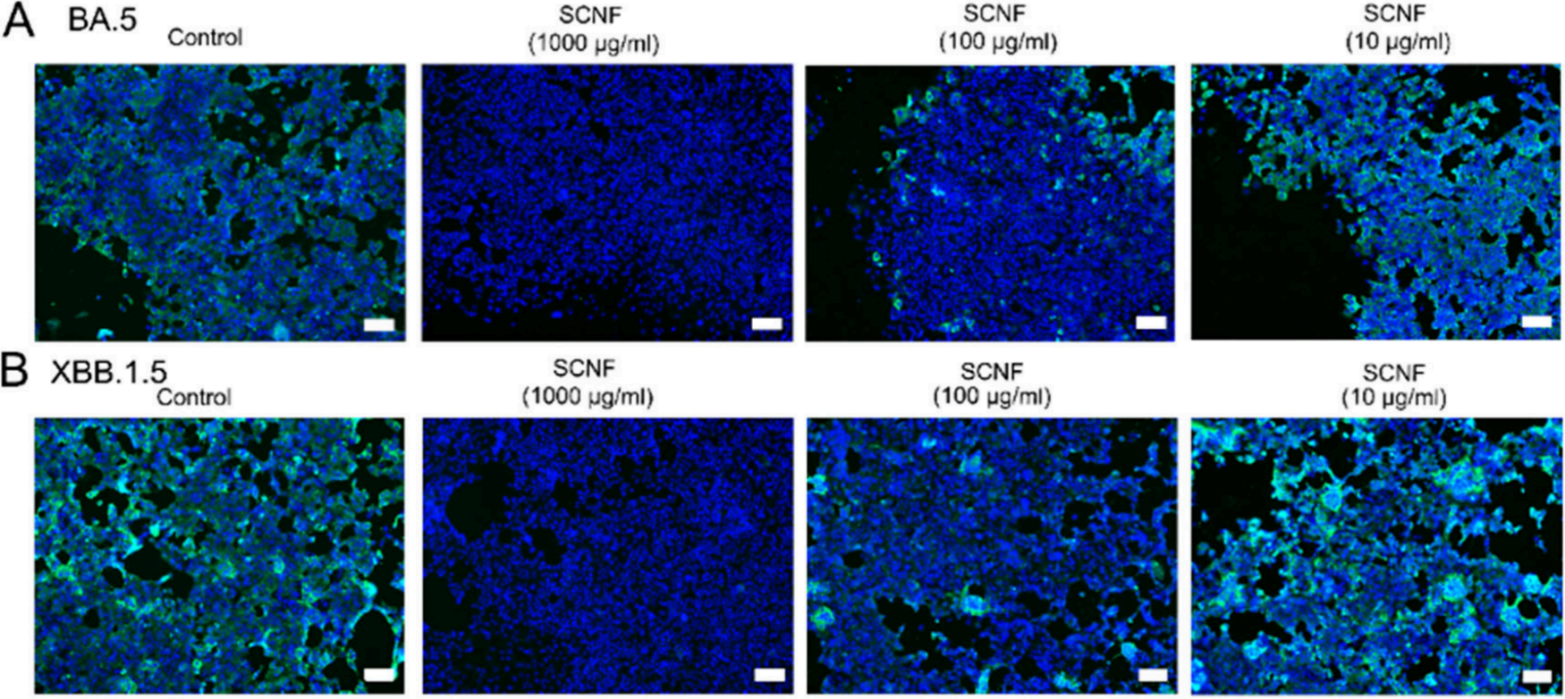
Immunofluorescent images revealing the infected cells in the preinfection inhibition assay toward (A) SARS-CoV-2 BA.5 and (B) XBB.1.5 variants. The cell nuclei are stained with Hoechst 33342 (blue signal), while the infected cells are stained by antibody against SARS-CoV-2 N protein (green signal). Scale bar = 100 μm.

As reported previously, polysulfate-based structures can inhibit the infection of by SARS-CoV-2 through electrostatic interactions with the receptor binding domain (RBD) of its spike protein.^36^ We therefore investigated the potential of SCNF-2 as a SARS-CoV-2 inhibitor. Similar to the experimental setting of HSV-1 infection inhibition, we preincubated the cells with the compounds and then infected them with SARS-CoV-2 omicron variants (BA.5 and XBB.1.5) at an MOI of 0.1. After 48 h, the infected cells were stained with antibodies against the SARS-CoV-2 N protein. In Figure 5 and Figure S12, we observed reduction of infected cells in the SCNF group (100 and 1000 μg/mL) compared to the control group and heparin group when incubated with SARS-CoV-2 BA.5 and XBB.1.5 variants, suggesting that SCNF can also inhibit the infection of SARS-CoV-2 and SCNF outperforms heparin on virus inhibition.

Then, microscale thermophoresis (MST) was used to determine the binding affinities (K_d_ values) of SCNF-2 against spike S1 ECD-His (K_d_ 199.1 μM) and Spike RBD-His (K_d_ 705.7 nM) (Table S1 and Figure S13). This could reflect that there may be a binding site in the RBD and another one in the greater S1 protein. According to the literature, the exterior surface of the SARS-CoV-2 virion is dominated by the spike protein filled with cationic domain, which is the binding site for SCNF through electrostatic interaction.^37,38^

## 4 Conclusion

In summary, a virus-binding mucus-mimetic Ca^2+^-SCNF hydrogel with a mucin-inspired composition and porous structure was synthesized. The SCNFs, with mucin-like structures, inhibited HSV-1 infection with similar activity as heparin. By simply introducing Ca^2+^ ions to SCNF solutions, mucus-like hydrogels were formed with the ability of preventing HSV-1 infection demonstrated by a transwell experiment. Additionally, SCNF was proven to inhibit SARS-CoV-2 infection. It is believed that the SCNF or Ca^2+^-SCNF hydrogel could be formulated into a nasal spray as a mucin or mucus mimetic to prevent virus infections, as shown in Figure S14.

Moreover, the synthesis of such a polymeric nanofibrous structure is straightforward and efficient and has a great potential for large scale production. Besides the biomedical applications, the SCNF may also be used as a substitute for conventional oxidized cellulose nanofiber in the production of novel biobased polymeric composites, such as highly transparent films and aerogels.

## Data Availability

The data that support the findings of this study are available in the Supporting Information of this article.

## Supporting Information Available

Video S1: Live-cell imaging was recorded over the period of 72 h/15 min to investigate the HSV-1 infection in the Vero E6 cells when incubated with Ca2+-SCNF hydrogel (MP4)
Video S2: Live-cell imaging was recorded over the period of 72 h/15 min to investigate the HSV-1 infection in the Vero E6 cells (MP4)
Video S3: Live-cell imaging was recorded over the period of 72 h/15 min to investigate the HSV-1 infection in the Vero E6 cells when incubated with Ca2+-OCNF hydrogel (MP4)
Experimental section; rheology tests of SCMC and SCNF; FTIR spectra of MCC, CMC, SCNF and OCNF; TEM image of OCNF; digital photograph of SCNF and OCNF; cellular toxicity of heparin and SCNFs; immunofluorescent images revealing the infected cells when incubated with heparin; virus inhibition ratios for CMC and MCC; digital photographs of K^+^-SCNF, Na^+^-SCNF, and H^+^-SCNF hydrogels; stability tests of representative 0.01 M Ca^2+^-SCNF overtime; SEM of Ca^2+^-SCNF hydrogel; scheme of titration of 0.01 M Ca^2+^-SCNF hydrogel precipitate after incubating with HSV-1; fluorescent images revealing the infected cells in transwell assay when incubated with Ca^2+^-OCNF hydrogel; immunofluorescent images revealing the infected cells toward SARS-CoV-2 BA.5 and XBB.1.5 variants after incubating with SCNF-2 (PDF)

## References

[ref1] Nathanson N. Chapter 1 - The Human Toll of Viral Diseases: Past Plagues and Pending Pandemics. In Viral Pathogenesis, Third ed.; Katze M. G., Korth M. J., Law G. L., Nathanson N., Eds.; Academic Press: Boston, 2016; pp 3–16.

[ref2] Chart Book: Tracking the Recovery From the Pandemic Recession. Center on Budget and Policy Priorities. https://www.cbpp.org/research/economy/tracking-the-recovery-from-the-pandemic-recession (accessed 2024/01/10/).

[ref3] COVID-19 Dashboard. John Hopkins University & Medicine. https://coronavirus.jhu.edu/map.html (accessed 2024/01/18/).

[ref4] Dogrammatzis C.; Waisner H.; Kalamvoki M. ″Non-Essential″ Proteins of HSV-1 with Essential Roles In Vivo: A Comprehensive Review. Viruses 2021, 13 (1), 17 10.3390/v13010017. PMC7824580 33374862

[ref5] Malik J. A.; Ahmed S.; Mir A.; Shinde M.; Bender O.; Alshammari F.; Ansari M.; Anwar S. The SARS-CoV-2 mutations versus vaccine effectiveness: New opportunities to new challenges. J. Infect Public Heal 2022, 15 (2), 228–240. 10.1016/j.jiph.2021.12.014. PMC8730674 35042059

[ref6] Ai X.; Wang D.; Honko A.; Duan Y.; Gavrish I.; Fang R. H.; Griffiths A.; Gao W.; Zhang L. Surface Glycan Modification of Cellular Nanosponges to Promote SARS-CoV-2 Inhibition. J. Am. Chem. Soc. 2021, 143 (42), 17615–17621. 10.1021/jacs.1c07798. 34647745

[ref7] Ghezzi S.; Cooper L.; Rubio A.; Pagani I.; Capobianchi M. R.; Ippolito G.; Pelletier J.; Meneghetti M. C. Z.; Lima M. A.; Skidmore M. A.; Broccoli V.; Yates E. A.; Vicenzi E. Heparin prevents Zika virus induced-cytopathic effects in human neural progenitor cells. Antivir Res. 2017, 140, 13–17. 10.1016/j.antiviral.2016.12.023. 28063994 PMC7113768

[ref8] Milewska A.; Zarebski M.; Nowak P.; Stozek K.; Potempa J.; Pyrc K. Human coronavirus NL63 utilizes heparan sulfate proteoglycans for attachment to target cells. J. Virol 2014, 88 (22), 13221–30. 10.1128/JVI.02078-14. 25187545 PMC4249106

[ref9] Vicenzi E.; Canducci F.; Pinna D.; Mancini N.; Carletti S.; Lazzarin A.; Bordignon C.; Poli G.; Clementi M. Coronaviridae and SARS-associated Coronavirus Strain HSR1. Emerg Infect Dis. 2004, 10 (3), 413 10.3201/eid1003.030683. 15109406 PMC3322807

[ref10] Sheng Y. H.; Hasnain S. Z. Mucus and Mucins: The Underappreciated Host Defence System. Front Cell Infect Microbiol 2022, 12, 856962 10.3389/fcimb.2022.856962. 35774401 PMC9238349

[ref11] Zanin M.; Baviskar P.; Webster R.; Webby R. The Interaction between Respiratory Pathogens and Mucus. Cell Host Microbe 2016, 19 (2), 159–68. 10.1016/j.chom.2016.01.001. 26867175 PMC4752725

[ref12] Koehler M.; Delguste M.; Sieben C.; Gillet L.; Alsteens D. Initial Step of Virus Entry: Virion Binding to Cell-Surface Glycans. Annu. Rev. Virol. 2020, 7 (1), 143–165. 10.1146/annurev-virology-122019-070025. 32396772

[ref13] Petrou G.; Crouzier T. Mucins as multifunctional building blocks of biomaterials. Biomater. Sci. 2018, 6 (9), 2282–2297. 10.1039/C8BM00471D. 30047553

[ref14] Pinzón Martín S.; Seeberger P. H.; Varón Silva D. Mucins and Pathogenic Mucin-Like Molecules Are Immunomodulators During Infection and Targets for Diagnostics and Vaccines. Front Chem. 2019, 7, 710 10.3389/fchem.2019.00710. 31696111 PMC6817596

[ref15] Bej R.; Haag R. Mucus-Inspired Dynamic Hydrogels: Synthesis and Future Perspectives. J. Am. Chem. Soc. 2022, 144 (44), 20137–20152. 10.1021/jacs.1c13547. 36074739 PMC9650700

[ref16] Bhatia S.; Lauster D.; Bardua M.; Ludwig K.; Angioletti-Uberti S.; Popp N.; Hoffmann U.; Paulus F.; Budt M.; Stadtmüller M.; Wolff T.; Hamann A.; Böttcher C.; Herrmann A.; Haag R. Linear polysialoside outperforms dendritic analogs for inhibition of influenza virus infection in vitro and in vivo. Biomaterials 2017, 138, 22–34. 10.1016/j.biomaterials.2017.05.028. 28550754

[ref17] Kwon S.-J.; Na D. H.; Kwak J. H.; Douaisi M.; Zhang F.; Park E. J.; Park J.-H.; Youn H.; Song C.-S.; Kane R. S.; Dordick J. S.; Lee K. B.; Linhardt R. J. Nanostructured glycan architecture is important in the inhibition of influenza A virus infection. Nat. Nanotechnol. 2017, 12 (1), 48–54. 10.1038/nnano.2016.181. 27775724

[ref18] Palika A.; Armanious A.; Rahimi A.; Medaglia C.; Gasbarri M.; Handschin S.; Rossi A.; Pohl M. O.; Busnadiego I.; Gübeli C.; Anjanappa R. B.; Bolisetty S.; Peydayesh M.; Stertz S.; Hale B. G.; Tapparel C.; Stellacci F.; Mezzenga R. An antiviral trap made of protein nanofibrils and iron oxyhydroxide nanoparticles. Nat. Nanotechnol. 2021, 16 (8), 918–925. 10.1038/s41565-021-00920-5. 34083772

[ref19] Bej R.; Nie C.; Ludwig K.; Ahmadi V.; Trimpert J.; Adler J. M.; Povolotsky T. L.; Achazi K.; Kagelmacher M.; Vidal R. M.; Dernedde J.; Kaufer B. B.; Haag R. Mucin-Inspired Single-Chain Polymer (MIP) Fibers as Potent SARS-CoV-2 Inhibitors. Angew. Chem., Int. Ed. 2023, 62 (29), e202304010 10.1002/anie.202304010. 37130003

[ref20] Tang S.; Puryear W. B.; Seifried B. M.; Dong X.; Runstadler J. A.; Ribbeck K.; Olsen B. D. Antiviral Agents from Multivalent Presentation of Sialyl Oligosaccharides on Brush Polymers. ACS Macro Lett. 2016, 5 (3), 413–418. 10.1021/acsmacrolett.5b00917. 35614714

[ref21] De Clercq E.; Li G. Approved Antiviral Drugs over the Past 50 Years. Clin Microbiol Rev. 2016, 29 (3), 695–747. 10.1128/CMR.00102-15. 27281742 PMC4978613

[ref22] Szymańska E.; Orłowski P.; Winnicka K.; Tomaszewska E.; Ba̧ska P.; Celichowski G.; Grobelny J.; Basa A.; Krzyżowska M. Multifunctional Tannic Acid/Silver Nanoparticle-Based Mucoadhesive Hydrogel for Improved Local Treatment of HSV Infection: In Vitro and In Vivo Studies. Int. J. Mol. Sci. 2018, 19 (2), 387 10.3390/ijms19020387. 29382085 PMC5855609

[ref23] Zhang Y.; Yao Q.; Xia C.; Jiang X.; Wang P. G. Trapping norovirus by glycosylated hydrogels: a potential oral antiviral drug. ChemMedChem. 2006, 1 (12), 1361–6. 10.1002/cmdc.200600135. 17042040

[ref24] Thongrom B.; Sharma A.; Nie C.; Quaas E.; Raue M.; Bhatia S.; Haag R. Scaffold Flexibility Controls Binding of Herpes Simplex Virus Type 1 with Sulfated Dendritic Polyglycerol Hydrogels Fabricated by Thiol-Maleimide Click Reaction. Macromol. biosci 2022, 22 (5), e2100507 10.1002/mabi.202100507. 35142052

[ref25] Hill D. B.; Vasquez P. A.; Mellnik J.; McKinley S. A.; Vose A.; Mu F.; Henderson A. G.; Donaldson S. H.; Alexis N. E.; Boucher R. C.; Forest M. G. A biophysical basis for mucus solids concentration as a candidate biomarker for airways disease. PloS one 2014, 9 (2), e87681 10.1371/journal.pone.0087681. 24558372 PMC3928107

[ref26] Jory M.; Bellouma K.; Blanc C.; Casanellas L.; Petit A.; Reynaud P.; Vernisse C.; Vachier I.; Bourdin A.; Massiera G. Mucus Microrheology Measured on Human Bronchial Epithelium Culture. Front. Phys. 2019, 7, 19 10.3389/fphy.2019.00019.

[ref27] Brunialti M.; Höfler T.; Nascimento M.; Trimpert J. Suicidal Phenotype of Proofreading-Deficient Herpes Simplex Virus 1 Polymerase Mutants. J. Virol 2023, 97 (1), e0135922 10.1128/jvi.01359-22. 36598203 PMC9888220

[ref28] Sirviö J. A.; Ukkola J.; Liimatainen H. Direct sulfation of cellulose fibers using a reactive deep eutectic solvent to produce highly charged cellulose nanofibers. Cellulose 2019, 26 (4), 2303–2316. 10.1007/s10570-019-02257-8.

[ref29] Gu J.; Catchmark J. M.; Kaiser E. Q.; Archibald D. D. Quantification of cellulose nanowhiskers sulfate esterification levels. Carbohydr. Polym. 2013, 92 (2), 1809–16. 10.1016/j.carbpol.2012.10.078. 23399223

[ref30] Yin C.; Shen X. Synthesis of cellulose carbamate by supercritical CO2-assisted impregnation: Structure and rheological properties. Eur. Polym. J. 2007 2007, 43 (5), 2111–2116. 10.1016/j.eurpolymj.2007.01.041.

[ref31] Kargarzadeh H. Handbook of Nanocellulose and Cellulose Nanocomposites; Wiley, 2017.

[ref32] Schroeder H. A.; Nunn K. L.; Schaefer A.; Henry C. E.; Lam F.; Pauly M. H.; Whaley K. J.; Zeitlin L.; Humphrys M. S.; Ravel J.; Lai S. K. Herpes simplex virus-binding IgG traps HSV in human cervicovaginal mucus across the menstrual cycle and diverse vaginal microbial composition. Mucosal Immunol 2018, 11 (5), 1477–1486. 10.1038/s41385-018-0054-z. 29988116 PMC6485947

[ref33] Tanaka M.; Kagawa H.; Yamanashi Y.; Sata T.; Kawaguchi Y. Construction of an excisable bacterial artificial chromosome containing a full-length infectious clone of herpes simplex virus type 1: viruses reconstituted from the clone exhibit wild-type properties in vitro and in vivo. J. virol 2003, 77 (2), 1382–91. 10.1128/JVI.77.2.1382-1391.2003. 12502854 PMC140785

[ref34] Chen Y.; Zhang L.; Yang Y.; Pang B.; Xu W.; Duan G.; Jiang S.; Zhang K. Recent Progress on Nanocellulose Aerogels: Preparation, Modification, Composite Fabrication, Applications. Adv. mater 2021, 33 (11), e2005569 10.1002/adma.202005569. 33538067 PMC11468492

[ref35] Bienenstock J.; Befus A. D. Mucosal immunology. Immunology 1980, 41 (2), 249–70. 7002769 PMC1458175

[ref36] Nie C.; Pouyan P.; Lauster D.; Trimpert J.; Kerkhoff Y.; Szekeres G. P.; Wallert M.; Block S.; Sahoo A. K.; Dernedde J.; Pagel K.; Kaufer B. B.; Netz R. R.; Ballauff M.; Haag R. Polysulfates Block SARS-CoV-2 Uptake through Electrostatic Interactions. Angew. Chem., Int. Ed. 2021, 60 (29), 15870–15878. 10.1002/anie.202102717. PMC8250366 33860605

[ref37] Nie C.; Sahoo A. K.; Netz R. R.; Herrmann A.; Ballauff M.; Haag R. Charge Matters: Mutations in Omicron Variant Favor Binding to Cells. Chembiochem: a European journal of chemical biology 2022, 23 (6), e202100681 10.1002/cbic.202100681. 35020256 PMC9015620

[ref38] Cotten M.; Phan M. V. T. Evolution of increased positive charge on the SARS-CoV-2 spike protein may be adaptation to human transmission. iScience 2023, 26 (3), 106230 10.1016/j.isci.2023.106230. 36845032 PMC9937996

