# Supplementary material for: Sulfated Cellulose Nanofiber Hydrogel with Mucus-Like Activities for Virus Inhibition
Source: ACS Appl Mater Interfaces. 2024 Nov 25;16(49):67504–13. doi: 10.1021/acsami.4c17998 (PMC11647751; doi:10.1021/acsami.4c17998)
Supplement: Supplementary file 4 — am4c17998_si_004.pdf [file am4c17998_si_004.pdf]

Supporting information

Sulfated cellulose nanofiber hydrogel with mucus-like activities for virus inhibition

Yanping Long,[a] *Mathias Dimde,*[a, c] *Julia M. Adler,*[b] *Ricardo Martin Vidal,*[b] *Tatyana L. Povolotsky,*[a] *Philip Nickl,*[a] *Katharina Achazi,*[a] *Jakob Trimpert,*[b] *Benedikt B. Kaufer,*[b] *Rainer Haag* [a,*], *Chuanxiong Nie* [a,*]

[a] Institute for Chemistry und Biochemistry, Freie Universität Berlin, Takustr. 3, 14195 Berlin, Germany

[b] Institut für Virologie, Freie Universität Berlin, Robert von Ostertag-Str. 7-13, 14163, Berlin, Germany

[c] Forschungszentrum für Elektronenmikroskopie und Gerätezentrum BioSupraMol, Freie Universität Berlin, Fabeckstraße 36A, 14195, Berlin, Germany

*E-mail: haag@zedat.fu-berlin.de; chuanxnie@zedat.fu-berlin.de

EXPERIMENTAL SECTION

Preparation of oxidized cellulose nanofibers (OCNF) derived from MCC. The synthesis of OCNF was performed according to the literature.[1] The TEMPO and NaBr were solubilized in deionized water, then NaClO and MCC were added to the above solution. Under constant stirring, 0.1 M HCl was used to adjust the pH to 10. During the reaction, 0.5 M NaOH solution was added dropwise to keep the pH at 10. The reaction was stopped when there was no pH change in 10 min by centrifugation. The OCNF was finally treated with water washing, dialysis, and lyophilization.

Cytotoxicity evaluations of SCNFs and heparin. Vero E6 cells (ATCC CRL-1586) were pre-seeded in 96-well plate and incubated at 37 ^{o}C and 5% CO_2. At the next day, cells were incubated with SCNFs and heparin of different concentrations for 24 h, afterwards, diluted CCK-8 solution (Sigma-Aldrich, Germany) was added to each well. After 2 h incubation, the mixture was detected by a microplate reader (Model 550, Bio-Rad) at 450 nm and analysed by a standard protocol to calculate the cellular toxicity as shown follows:

$$\text{Cell viability (\%)} = \left(\frac{\text{ABS450 (compounds)} - \text{ABS450 (negative control)}}{ABS450\ (positive\ control)}\right) \times 100\%$$

Microscale thermophoresis (MST) tests. Binding affinities were determined via MST using the Monolith NT.115Pico instrument with premium capillaries (SKU: MO-K025) from Nanotemper. The proteins used in this study were Spike S1-His (Cat# 40591-V08H) and Spike RBD-His (Cat# 40592-V08H) of the Sars-CoV-2 (2019-nCoV) virus (Wuhan strain) was obtained from SinoBiological and labeled using the protein labeling kit Red-NHS 2nd generation (SKU: MO-L011) from Nanotemper following the provided protocol. The final concentration of the labeled protein was determined using the Nanodrop instrument (ThermoFisher Scientific). The protocol as described is as

follows:[2] briefly the tested SCNF was serially 2-fold diluted in DPBS with 0.05% (v/v) Tween (PBST) with a resulting volume of 6 μL. 6 μL of S1 protein at a concentration of 8 mM was added to the mixture resulting in a final volume of 12 μL. The final mixture of PBST, SCNF and labeled S1 was vortexed, spun down and equilibrated at room temperature for 60 seconds before collecting the mixtures in premium capillaries for measurement. MST measurements were performed at 22 ^{o} C for MST over a 30 s time interval using the MO.Control software (Nanotemper) at 20% Red-LED excitation and 20% IR-laser power. The resultant data were analyzed via Affinity Analysis Software v2.3 (Nanotemper), exported and plotted with Prism 10.0.2 (Graphpad by Dotmatics).

SARS-CoV-2 inhibition of SCNF. The SARS-CoV-2 inhibition of SCNF was studied by pre-infection inhibition assay. Briefly, Vero E6 cells were seeded in 96-well plates. After being washed with PBS and refilled with 90 µL of infection medium (MEM, 1% FBS, P/S), 10 uL of samples were added, diluted 10 times in the medium and incubated on the cells for 90 min. Finally, 10 µL of virus dilution (MOI = 0.1) was added to each well and the plates were incubated at 37 ^{o}C for 45 min. Subsequently, medium was removed and cells were washed once with PBS. Infection medium was replenished to 100 µL and cells were incubated at 37 ^{o}C for 48 h. After 48 h, cells were washed and fixed with 4%PBS-buffered formaldehyde was added for 24 h.

To stain the infected cells, the fixed cells were permeabilized in 0.1% Triton X-100, followed by a blocking step with 5% BSA for 30 min with slight shaking. Subsequently, the infected cells were incubated overnight with primary antibody, SARS-CoV-2 Nucleocapsid Monoclonal Antibody (Invitrogen). After being washed, cells were incubated with secondary antibody, Goat-anti-mouse IgG Alexa Fluor 488 (Invitrogen). Finally, the cell nuclei were stained by Hoechst 33342 and the cells were washed once and kept in PBS for imaging.

Supporting Figures

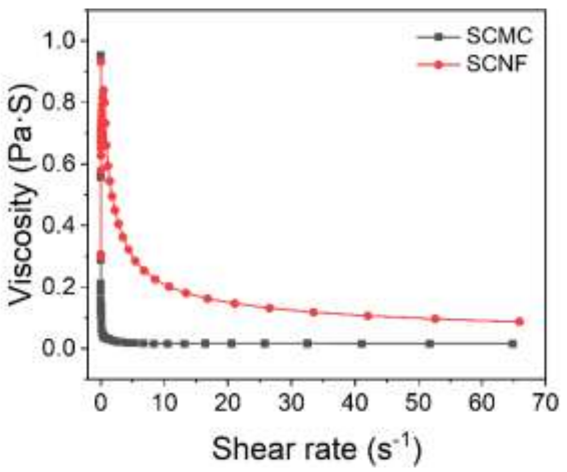

Figure S1. Shear rate-dependent viscosity moduli of pure SCMC and SCNF.

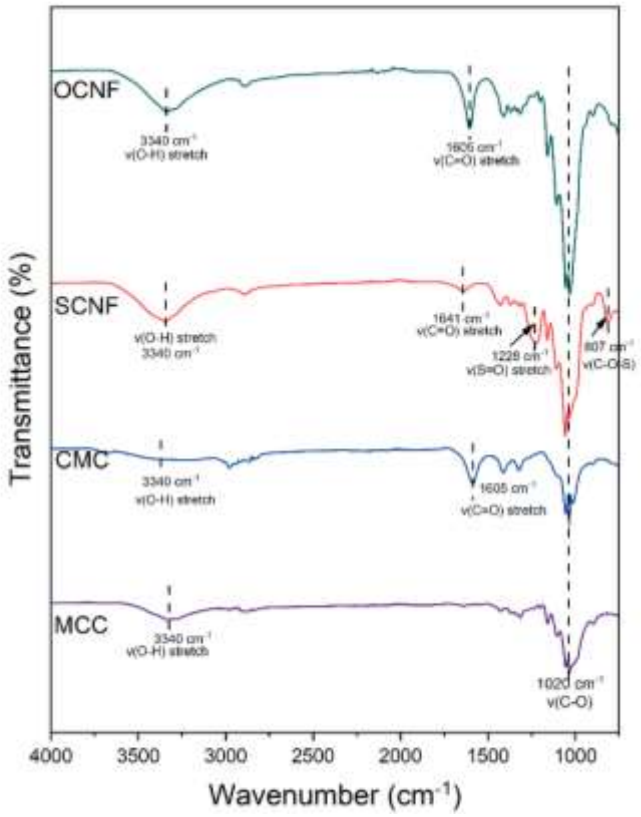

Figure S2. FTIR spectra of MCC, CMC, SCNF and OCNF.

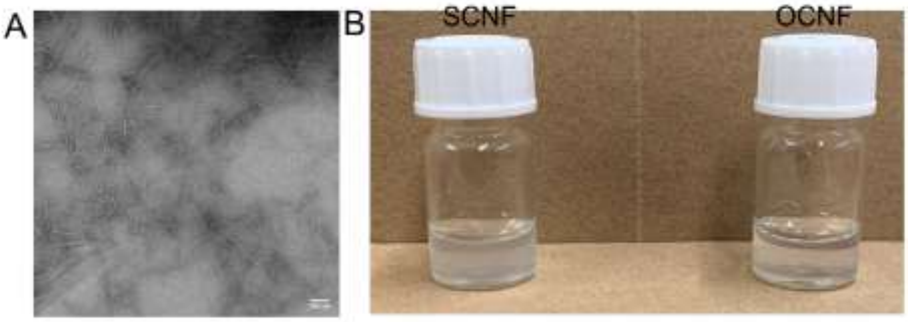

Figure S3. (A) TEM image of OCNF. Scale bar: 100nm. (B) Digital photograph of SCNF and OCNF.

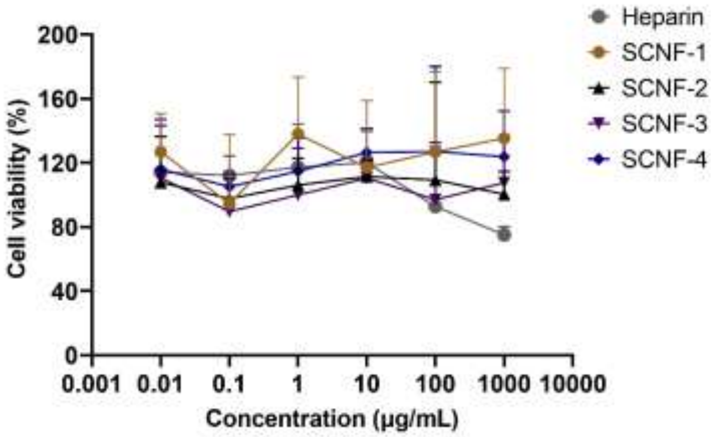

Figure S4. Evaluation of cellular toxicity of heparin and SCNFs incubated with Vero E6 cells. Values are expressed as mean ±SD, n=3.

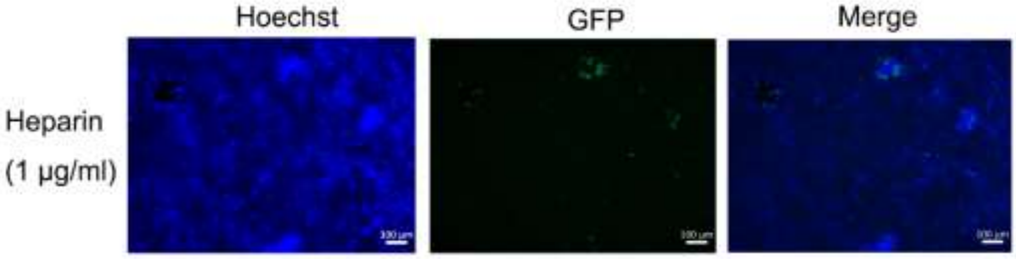

Figure S5. Representative immunofluorescent images revealing the infected cells in the pre-infection inhibition assay. The cell nuclei are stained by Hoechst in blue channel, while the infected cells are presented in green channel.

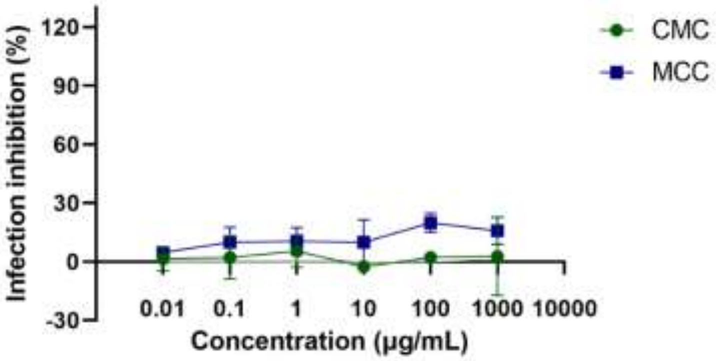

Figure S6. Inhibition ratios for the different cellulose. Values are expressed as mean ±SD, n=3.

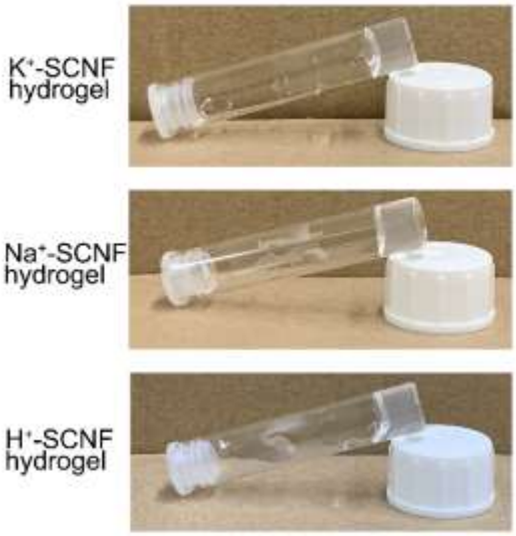

Figure S7. Digital photographs of K^+-SCNF, Na^+-SCNF and H^+-SCNF hydrogels. The concentration of K^+, Na^+ and H^+ is 0.1 M.

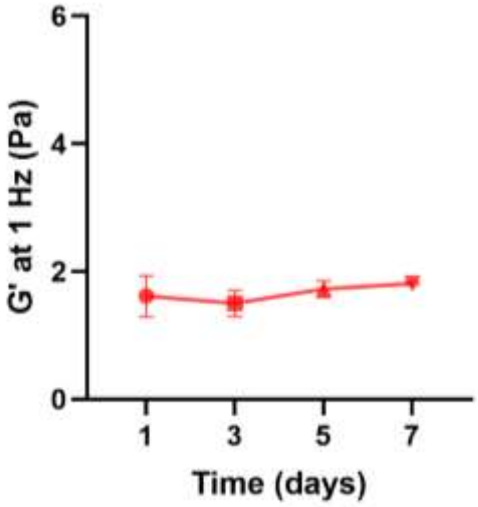

Figure S8. Stability tests of representative 0.01 M Ca^{2+}-SCNF overtime.

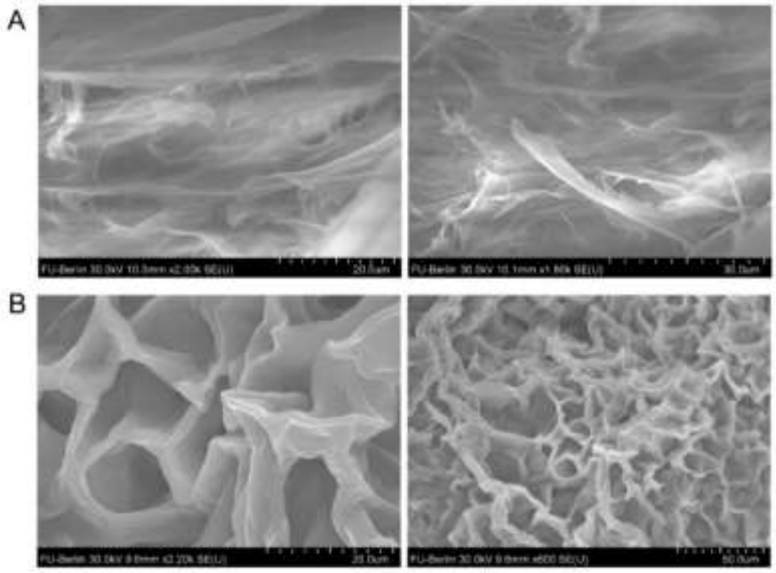

Figure S9. SEM of Ca^{2+}-SCNF hydrogel in the presence of (A) 0.01 M Ca^{2+} and (B) 0.1 M Ca^{2+} after being freeze-dried. Apparently, the 0.1 M Ca^{2+}-SCNF hydrogel presented more compact morphology than 0.01 M Ca^{2+}-SCNF hydrogel.

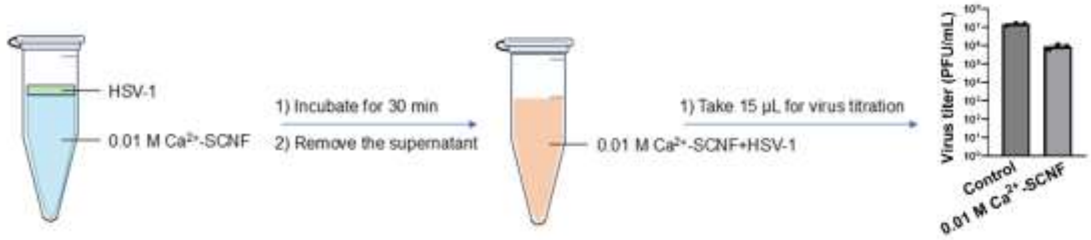

Figure S10. Titration of 0.01 M Ca^{2+}-SCNF hydrogel precipitate after incubating with HSV-1. Values are presented as mean ± SD, n=3.

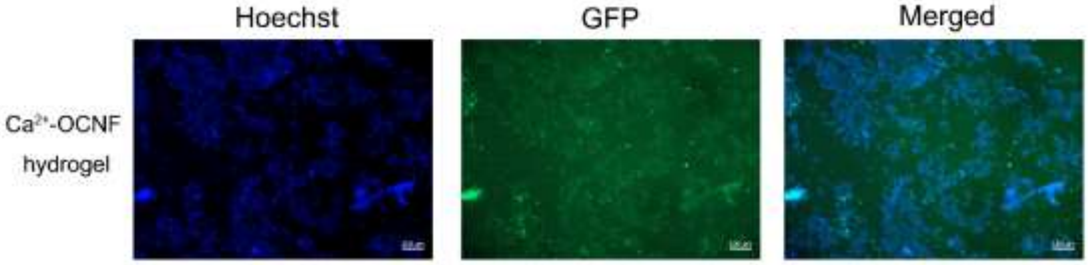

Figure S11. Representative fluorescent images revealing the infected cells in transwell assay when incubated with Ca^{2+}-OCNF hydrogel. The cell nuclei are stained by Hoechst in blue channel, while the infected cells are presented in green channel.

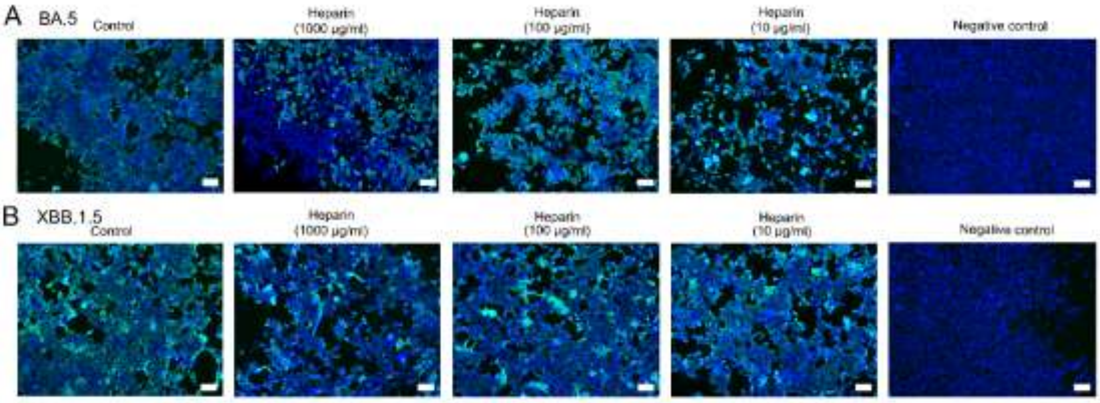

Figure S12. Immunofluorescent images revealing the infected cells in the pre-infection inhibition assay towards (A) SARS-CoV-2 BA.5 and (B) XBB.1.5 variants. The cell nuclei are stained by Hoechst in blue channel, while the infected cells are stained by antibody against SARS-CoV-2 N protein in green channel.

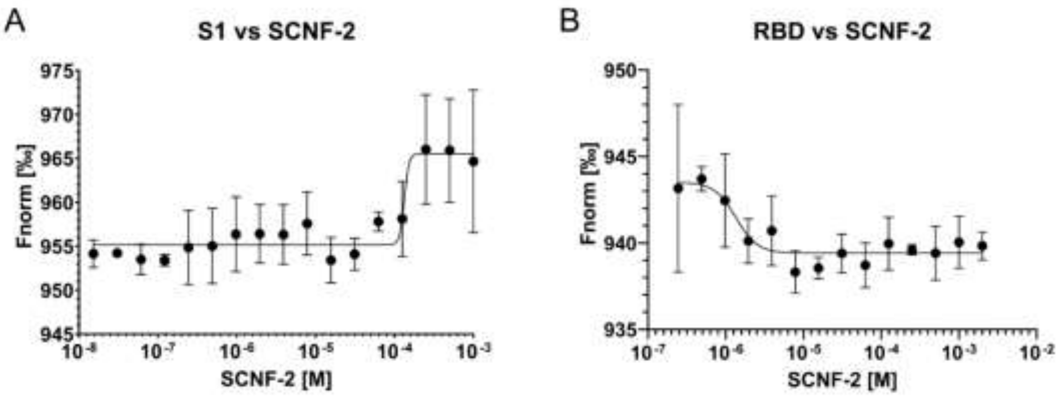

Figure S13. Microscale thermophoresis measurements of A) SCNF-2 against Spike S1 ECD-His, B) SCNF-2 against Spike RBD-His. Each data point represents biological repeats of at least N=4. Data points were fitted with one sited fit assuming a 1:1 ligand to receptor ratio.

Table S1. Summary of binding affinities (K_d) using microscale thermophoresis (MST). N is the number of biological repeats for each binding partner comparison.

	SCNF-2
S1	K_d =199.1 µM ±121.6 µM (N=6)
RBD	K_d =705.7 nM ±578.4 nM (N=4)

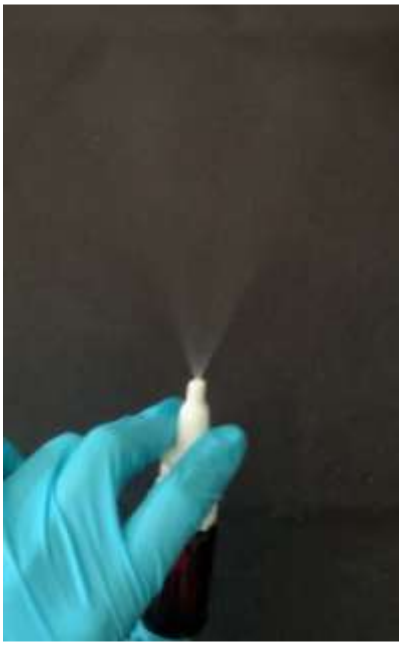

Figure S14 Potential nasal spray application via using SCNF-2.

Supporting Videos

Video S1. Live-cell imaging was recorded over the period of 72 h/15 min to investigate the HSV-1 infection in the Vero E6 cells when incubated with Ca^{2+}-SCNF hydrogel.

Video S2. Live-cell imaging was recorded over the period of 72 h/15 min to investigate the HSV-1 infection in the Vero E6 cells.

Video S3. Live-cell imaging was recorded over the period of 72 h/15 min to investigate the HSV-1 infection in the Vero E6 cells when incubated with Ca^{2+}-OCNF hydrogel.

References:

(1) Wu, W.; Lu, Z.; Lu, C.; Sun, X.; Ni, B.; Cölfen, H.; Xiong, R., Bioinspired Stabilization of Amorphous Calcium Carbonate by Carboxylated Nanocellulose Enables Mechanically Robust, Healable, and Sensing Biocomposites. *ACS Nano* **2023,** *17* (7), 6664-6674.
(2) Page, T. M.; Nie, C.; Neander, L.; Povolotsky, T. L.; Sahoo, A. K.; Nickl, P.; Adler, J. M.; Bawadkji, O.; Radnik, J.; Achazi, K.; Ludwig, K.; Lauster, D.; Netz, R. R.; Trimpert, J.; Kaufer, B.; Haag, R.; Donskyi, I. S., Functionalized Fullerene for Inhibition of SARS-CoV-2 Variants. *Small* **2023,** *19* (15), 2206154.
